# PF-03882845, a non-steroidal mineralocorticoid receptor antagonist, prevents renal injury with reduced risk of hyperkalemia in an animal model of nephropathy

**DOI:** 10.3389/fphar.2013.00115

**Published:** 2013-10-14

**Authors:** Stephen Orena, Tristan S. Maurer, Li She, Rena Eudy, Vincent Bernardo, Darla Dash, Paula Loria, Mary E. Banker, Meera Tugnait, Carlin V. Okerberg, Jessie Qian, Carine M. Boustany-Kari

**Affiliations:** Pfizer Groton Research and DevelopmentGroton, CT, USA

**Keywords:** urinary albumin to creatinine ratio, UACR, nephropathy, albuminuria, hyperkalemia, mineralocorticoid receptor

## Abstract

The mineralocorticoid receptor (MR) antagonists PF-03882845 and eplerenone were evaluated for renal protection against aldosterone-mediated renal disease in uninephrectomized Sprague-Dawley (SD) rats maintained on a high salt diet and receiving aldosterone by osmotic mini-pump for 27 days. Serum K^+^ and the urinary albumin to creatinine ratio (UACR) were assessed following 14 and 27 days of treatment. Aldosterone induced renal fibrosis as evidenced by increases in UACR, collagen IV staining in kidney cortex, and expression of pro-fibrotic genes relative to sham-operated controls not receiving aldosterone. While both PF-03882845 and eplerenone elevated serum K^+^ levels with similar potencies, PF-03882845 was more potent than eplerenone in suppressing the rise in UACR. PF-03882845 prevented the increase in collagen IV staining at 5, 15 and 50 mg/kg BID while eplerenone was effective only at the highest dose tested (450 mg/kg BID). All doses of PF-03882845 suppressed aldosterone-induced increases in collagen IV, transforming growth factor-β 1 (*Tgf-β 1*), interleukin-6 (*Il-6*), intermolecular adhesion molecule-1 (*Icam-1*) and osteopontin gene expression in kidney while eplerenone was only effective at the highest dose. The therapeutic index (TI), calculated as the ratio of the EC_50_ for increasing serum K^+^ to the EC_50_ for UACR lowering, was 83.8 for PF-03882845 and 1.47 for eplerenone. Thus, the TI of PF-03882845 against hyperkalemia was 57-fold superior to that of eplerenone indicating that PF-03882845 may present significantly less risk for hyperkalemia compared to eplerenone.

## Introduction

Mounting clinical evidence suggests a key role for aldosterone in the progression of nephropathy. Inhibitors of the renin-angiotensin-aldosterone system (RAAS), such as angiotensin receptor blockers (ARB) and angiotensin-converting enzyme inhibitors (ACEI) have been demonstrated to delay the onset of macroalbuminuria or frank nephropathy in diabetics (Brenner et al., 2001; Lewis et al., 2001; Parving et al., 2001; Remuzzi et al., 2002; Ruggenenti et al., 2004). Nonetheless, a significant proportion of patients continue to progress to macroalbuminuria and overt nephropathy (Parving et al., 2001; Ruggenenti et al., 2004). Many patients on ACEI or ARB treatment exhibit “aldosterone escape,” whereby the initial response of decreased aldosterone and urinary albumin to creatinine ratio (UACR) is followed by a rebound of aldosterone and reversion of UACR to pre-treatment levels (Lijnen et al., 1982; Pitt, 1995). The mineralocorticoid receptor (MR) antagonist spironolactone effectively reduced UACR in diabetic nephropathy patients exhibiting “aldosterone escape” while on ACEI therapy (Sato et al., 2003). Moreover, eplerenone, another marketed MR antagonist, decreased UACR in type 2 diabetics with mild to moderate nephropathy to a greater extent than that achieved with ACEI treatment alone, and independently of changes in blood pressure (Epstein, 2006; Epstein et al., 2006). However, inhibition of the RAAS with ACEI, ARB, direct renin inhibitors (DRI) or MR antagonists especially in dual or triple combination, increased the risk of hyperkalemia in patients with heart failure (HF), or chronic kidney disease (McMahon, 2001; Sica, 2002; Palmer, 2004; Weir and Rolfe, 2010).

Aldosterone regulates Na^+^ and K^+^ balance and hence extracellular fluid volume and blood pressure (Levy et al., 2004; Muldowney et al., 2009). Under conditions of low blood volume or reduced renal perfusion, plasma angiotensin II (angII) and K^+^ levels increase and synergistically stimulate aldosterone secretion from the adrenals thereby promoting Na^+^ and water retention in the proximal tubule and driving sodium-dependent K^+^ secretion in the distal tubule of the kidney (Palmer, 2004). Thus, when renal filtration is severely compromised, as in patients with chronic kidney disease (CKD), cardiometabolic syndrome or HF, Na^+^ reabsorption in the proximal tubule becomes highly efficient. Under these conditions, urinary volume and Na^+^ delivery to the distal tubule become rate limiting for Na^+^-dependent K^+^ secretion and hyperkalemia may ensue (Palmer, 2004). Plasma aldosterone is increased in some patients with CKD (Berl et al., 1978; Hene et al., 1982), diabetes (Hollenberg et al., 2004), and metabolic syndrome (Bochud et al., 2006), and plasma aldosterone correlates positively with UACR (Bianchi et al., 2005) and negatively with glomerular filtration rates (GFR) in CKD patients (Quinkler et al., 2005). However, and given the pivotal role of aldosterone in regulating K^+^ excretion, the beneficial UACR lowering effect of MR antagonists may be offset by an increased risk of hyperkalemia. Therefore, a MR antagonist that decreases UACR without increasing the risk of hyperkalemia would be highly desirable for the treatment and prevention of diabetic nephropathy.

PF-03882845 is a selective and potent non-steroidal MR antagonist. To determine whether PF-03882845 differentiates from traditional steroidal MR antagonists, we sought to investigate the effect of PF-03882845 on UACR and electrolyte balance in preclinical studies. The concentration effect curves for the prevention of increases in UACR and for elevation of serum K^+^ were established for PF-03882845 and eplerenone in a rat model of aldosterone-induced renal damage. Additionally, the effect of the compounds on urinary Na^+^/K^+^ ratio, a surrogate biomarker for electrolyte disturbances, was investigated. Our data indicate a larger therapeutic index, calculated as the ratio of the EC_50_ for increasing serum K^+^ or urinary Na^+^/K^+^ to the EC_50_ for UACR lowering, for PF-03882845 compared to eplerenone.

## Materials and methods

All procedures were conducted in accordance with Institutional Animal Care and Use Committee (IACUC) guidelines and regulations at Pfizer Inc (Groton, CT).

### Selectivity and potency of PF-03882845 and eplerenone

Huh7 cells were transiently transfected with a luciferase reporter gene under the control of a Gal4 response element (Gal4-RE-luc) and a plasmid containing the Gal4 DNA binding domain fused to MR ligand binding domain (Gal4-MR-LBD). Transfected cells were treated for 24 h with the EC_80_ concentration of activating ligand in the presence or absence of PF-03882845 or eplerenone in serum free medium. Luciferase activity was measured and the IC_50_ determined for each compound.

### Effect of chronic administration of eplerenone and PF-03882845 on aldosterone-mediated renal injury

#### Study A

Fifty five male uninephrectomized Sprague Dawley (SD) rats (Charles River, MA) were singly housed and provided food and water *ad-libitum*. Following an acclimation period, baseline 24 h urine samples were collected and analyzed for albumin and creatinine measurement. Baseline blood was taken by tail bleed for measurement of serum K^+^. Rats were then randomized into 5 groups. Four groups of rats (*n* = 11 per group) were implanted subcutaneously (SC) with Alzet mini-pumps (Alzet, Cupertino, CA) to deliver 2.5 μ L/h aldosterone (0.75 μ g/h) in 0.01% dimethylsulfoxide (DMSO) under isoflurane anesthesia. One group was sham operated (Veh Sham, *n* = 11). Immediately post-surgery, all rats were switched to a 6% high salt diet (Teklad TD.90230) and water containing 0.3% KCl *ad-libitum*. The following day, treatment with eplerenone was initiated in the 4 groups of rats that were implanted with aldosterone mini-pumps. Rats received either vehicle (Veh Aldo), or one of 3 doses of eplerenone: 15, 50, or 450 mg/kg BID by oral gavage. The sham-operated group also received vehicle (Veh Sham). Dosing was performed at 6 am and 4 pm for a duration of 27 days. Eplerenone was dissolved in vehicle comprised of 0.5% methyl cellulose, 0.1% polysorbate 80 in water.

#### Study B

For study B the same protocol as study A was used, but PF-03882845 was administered in lieu of eplerenone at doses of 5, 15, and 50 mg/kg BID by oral gavage. PF-03882845 was dissolved in vehicle comprised of 0.5% methyl cellulose, 0.1% polysorbate 80 in water.

### Urine collection

Twenty-four hours urine samples were collected at baseline, on Days 14 and 25 of treatment. Urine volume was recorded and samples were utilized for creatinine and albumin concentrations.

### Plasma/serum collection

Rats were bled at baseline for plasma K^+^ measurement. Following the first dose of the respective compounds, rats were bled at 1, 2, 4, 7 h for drug levels measurements. On Day 14 of treatment, rats were bled at 7 h post the first dose of the day for plasma drug levels and serum K^+^ measurements. Additionally on Day 26, rats were bled at 1, 2, 4, 7 h post-dose for plasma drug levels measurement. On Day 27 (at necropsy, ~16 h post final dose), rats were bled for measurement of serum K^+^ and plasma drug levels measurements.

### Tissue collection

On Day 27 rats were euthanized with CO_2_, in accordance with IACUC guidelines and regulations at Pfizer Inc (Groton, CT). Kidney was removed, and weighed. A section of kidney was fixed in 10% formalin for histological analysis and a second portion of kidney (200 mg) was stored in a RNA stabilization reagent (RNAlater, Qiagen) for gene expression analysis.

### Histology

Kidney sections were stained with a rabbit anti-collagen-IV antibody (Thermo Scientific) and detected with an anti-rabbit antibody conjugated to horseradish peroxidase (HRP). Slides were counterstained with hematoxylin. Data were expressed as percent collagen IV staining of the kidney cortex.

### RT-PCR

Kidney samples (~30 mg) were removed from RNAlater and homogenized in 700 μ L of buffer RLT for RNA purification using an RNeasy Mini Kit (Qiagen) with on-column DNase treatment. Purified RNA was quantitated by NanoDrop. Samples (20 ng) were analyzed by RT-PCR to assess relative mRNA expression of Collagen-IV (*Col4a1*), osteopontin (*Spp1*), interleukin-6 (*Il-6*), intercellular adhesion molecule 1 (*Icam-1*), monocyte chemoattractant protein-1 (*Mcp-1*) and transforming growth factor β-1 (*Tgf-β 1*) using gene expression assays (Applied Biosystems), RNA to Ct Kit (Applied Biosystems) and the SDS-7900HT (Applied Biosystems). Expression data were normalized to mRNA expression of the endogenous control hypoxanthine phosphoribosyltransferase 1 (*Hprt1*). Data were expressed as relative quantitation (RQ) units using Veh Sham animal No. 2 as comparator (ie RQ equal to “1”).

### Biomarker measurements

Serum K^+^, urinary albumin and creatinine were measured using the Siemens Advia 2400 Chemistry Analyzer and reagents from Siemens Healthcare Diagnostics (Washington, DC). UACR was calculated as the ratio of urinary albumin concentration to creatinine concentration. Plasma osteopontin was measured on day 27 samples using an enzyme immunoadsorbent assay (Enzo Life Sciences, Inc., Farmingdale NY).

### Compound exposure

Plasma was analyzed for eplerenone or PF-03882845 using a non-GLP LC/MS/MS method. All calculations were performed in Watson LIMS (v7.2, Thermo, Inc, Philadelphia, PA) and individual data were used in all pharmacokinetic analyses. The area under the plasma concentration-time curve (AUC(0-last)) was estimated using the linear trapezoidal rule.

### Pharmacokinetic/pharmacodynamic (PK-PD) modeling for study A and study B

Population PK-PD modeling was conducted in NONMEM (version VI 2.0; GloboMax LLC. Hanover, MD). Analysis was conducted in a piece-wise manner whereby individual pharmacokinetic parameters estimated for each animal in step 1 were fixed during estimation of pharmacodynamic parameters in the second step.

The pharmacokinetics of eplerenone and PF-03882845 were characterized with one and two compartmental models, respectively. In both cases first order absorption and elimination was assumed. Decreases in exposure upon multiple dosing for both compounds were characterized assuming that clearance was induced between Days 1 and 7 and remained constant thereafter. Population variance on the rate of absorption, elimination and volume of distribution were assumed in order to provide the best possible description of exposure in individual animals. Individual pharmacokinetic parameter estimates were carried forward in modeling the PK-PD relationship to UACR and serum K^+^.

Increases in UACR in vehicle controls were modeled with an indirect response model where the initial value (i.e., UACR at the start of the study) represents a non-steady state condition that varies between individual rats. Drug effect was parameterized such that the increase in UACR was inhibited via an indirect response *I*_max_ model (Equation 1). Both eplerenone and PF-03882845 PK-PD data were modeled simultaneously. The *I*_max_, *K*_in_, and *K*_out_ parameters were assumed to be drug independent and only the IC_50_ parameter was assumed to be drug dependent.

(1) $$\frac{dUACR}{dt}=K_{\text{in}}\times \left[1-\frac{I_{\text{max}}\times C}{C+IC_{50}}\right]-K_{\text{out}}\times \text{UACR}$$

For modeling purposes, serum K^+^ data were transformed according to Equation 2. This formula allows for the calculation of drug-induced changes in serum K^+^ (from baseline), which are above and beyond that measured in the vehicle group. T × T and T × 0 represent the measured serum K^+^ in each drug treated individual at times equal to “T” and 0, respectively. VehT and Veh0 represent the mean measured serum K^+^ in the vehicle group at times equal to “T” and 0, respectively.

(2) ΔserumK=(T×T−T×0)−(VehT-Veh0)

The relationship between drug exposure and Δ serum K^+^ was modeled using a modified indirect response model whereby drug creates a concentration-dependent input rate (Equation 3). Both eplerenone and PF-03882845 PK-PD data were modeled simultaneously. The *K*_in, max_, and *K*_out_ parameters were assumed to be drug independent and only the EC_50_ parameter was assumed to be drug dependent.

(3) $$\begin{array}{l} \frac{d\Delta \text{serum}K}{dt}=K_{\text{in}}-K_{\text{out}}\times \Delta \text{serum}K;\text{where }K_{\text{in}}\\ \;=\frac{K_{\text{in},\text{ max}}\times C}{C+EC_{50}} \end{array}$$

### Acute effect of MR antagonists on urinary Na^+^/K^+^ ratio

Male, carotid artery cannulated, Sprague Dawley (SD) rats between 11 and 12 weeks of age were obtained (Charles River, MA), singly housed and acclimated in metabolic chambers for one week prior to the start of the experiment. Water and food (Purina 5001 pellets) were provided *ad-libitum* throughout the study. In one study, rats were randomly assigned to receive either vehicle (0.5% methyl cellulose, 0.1% polysorbate 80) alone or eplerenone at one of 3 doses: 5, 30 or 300 mg/kg. In a second study, rats were randomly assigned to receive either vehicle (0.5% methyl cellulose, 0.1% polysorbate 80) or PF-03882845 at one of 3 doses: 3, 10 and 30 mg/kg. On the evening prior to treatment administration, baseline urine was collected from 4 pm until 8 am on the day of treatment. Animals were then orally gavaged with their respective treatments at 8 am and urine collected at intervals of 0–2, 2–4, and 4–7 h post dose. After collection, urine was stored at −80°C until analyzed. Baseline blood was collected from carotid artery cannulas prior to dosing, and at 1, 2, 4, and 7 h post dose. Samples were centrifuged and plasma was collected and stored at −80°C for compound exposure measurements using the methodology described above. Urine sodium and K^+^ were measured using the Siemens Advia 2400 Chemistry Analyzer with reagents from Siemens Healthcare Diagnostics. Data were expressed as urinary Na^+^/K^+^ ratio.

### PK modeling for eplerenone

A two compartment distribution model with zero order absorption was used (1 h duration = observed Tmax). Clearance (CL) was estimated as a function of dose:

$$\text{CL}={\left(\text{THETA}{\left(1\right)}^{*}{\left(30/\text{DOSE}\right)}^{**}\text{PWR}\right)}^{*}\text{EXP}(\text{ETA}(1))$$

where Theta(1) is CL at 30 mg/kg, and PWR is a power function allowing non-linear change in CL with dose. Conditional estimates of CL, V1, and Q were obtained. A proportional residual error model was used.

### PK modeling for PF-03882845

A one compartment distribution model with first order absorption was used. Conditional estimates of KA, CL, and V1 were obtained. A proportional residual error model was used.

### PK/PD modeling of urinary Na^+^/K^+^

A two compartment linear model with first order absorption was used for both drugs. Conditional PK parameter estimates were read in with the input data. For PF-03882845, drug was dosed into compartment 1 (depot); Q was fixed to 0 and V2 was fixed to 1. For eplerenone, KA was fixed to 1 and drug was “infused” into compartment 2 over 1 h. The PD endpoint was the “double delta” in urinary Na^+^/K^+^ ratio (measured at the midpoint of the urine collection interval). Mean vehicle Na^+^/K^+^ ratio at time = 0 (Veh0) and at each urine collection interval (VehT) was estimated using vehicle data from both drug studies combined. Individual animal Na^+^/K^+^ ratio observed at time = 0 (T × 0) and at the midpoint of collection intervals (T × T) were used to calculate the “double delta” change in Na^+^/K^+^ ratio as follows:

DDresponse=(TxT-Tx0)−(VehT-Veh0)

Using this approach each animal's time zero response = 0.

### Statistical analyses

UACR and serum K^+^ data were analyzed using a mixed ANOVA model accounting for repeated measures where treatment group, days post operation and treatment day interaction effect were introduced as a fixed factor whereas the animal factor was considered as a random effect nested in the treatment factor. The baseline value for each animal was introduced as a covariate. The primary comparisons were vehicle sham group vs. the other four groups and the vehicle aldosterone group vs. treatment groups. A two-sided 5% test was conducted.

For Study A and Study B, kidney gene expression data, collagen IV staining data and plasma osteopontin data were analyzed with a One-Way ANOVA and a Tukey post test using GraphPad Prism 5. When unequal variances were observed, data were log-transformed prior to analysis. The primary comparisons were vehicle sham group vs. the other four groups and the vehicle aldosterone group vs. treatment groups.

## Results

### Potency of PF-03882845 and eplerenone

PF-03882845 exhibited a greater potency compared to eplerenone in a serum free functional reporter assay utilizing the human MR ligand binding domain [PF-03882845 geometric mean IC_50_ = 0.755 nM (90% confidence interval: 0.501–1.11 nM; *n* = 6), eplerenone IC_50_ = 109 nM (90% confidence interval: 79.3–150 nM; *n* = 6)].

### Effect of eplerenone and PF-03882845 on UACR and serum K^+^

**Figure 1:**
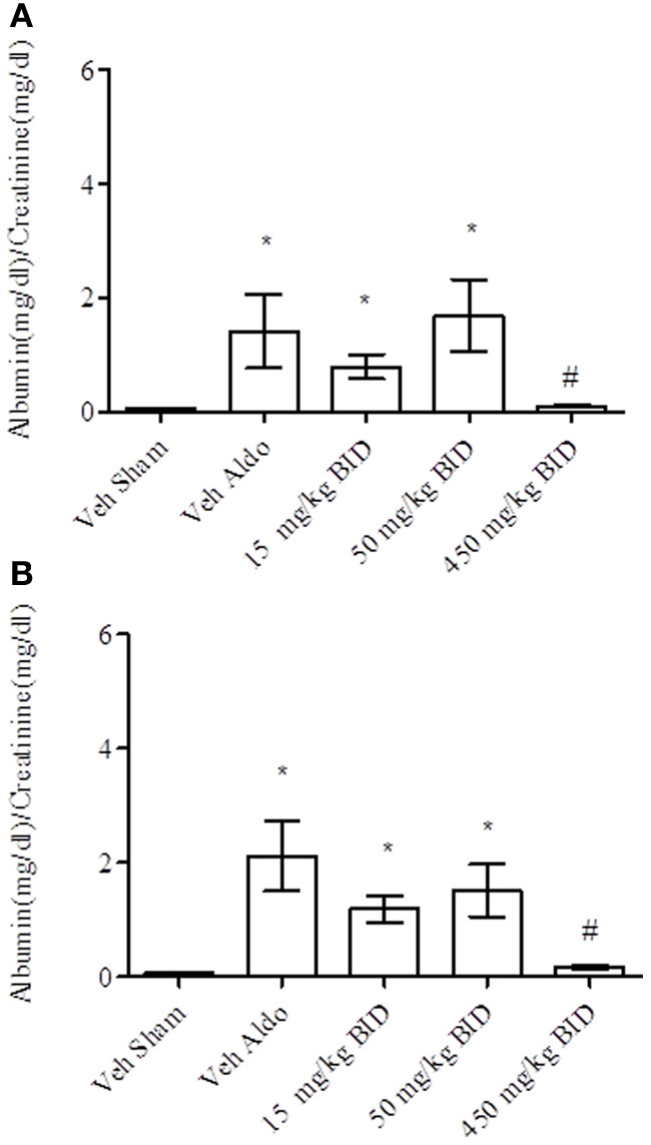
**Effect of eplerenone on UACR on day 14 (A) and day 26 (B).** Data are represented as mean ± standard error of the mean. See Materials and Methods for statistical analysis. ^*^Indicates a significant difference from the vehicle sham (Veh Sham) group; ^#^Indicates a significant difference from the vehicle + aldosterone (Veh Aldo) group.

**Figure 2:**
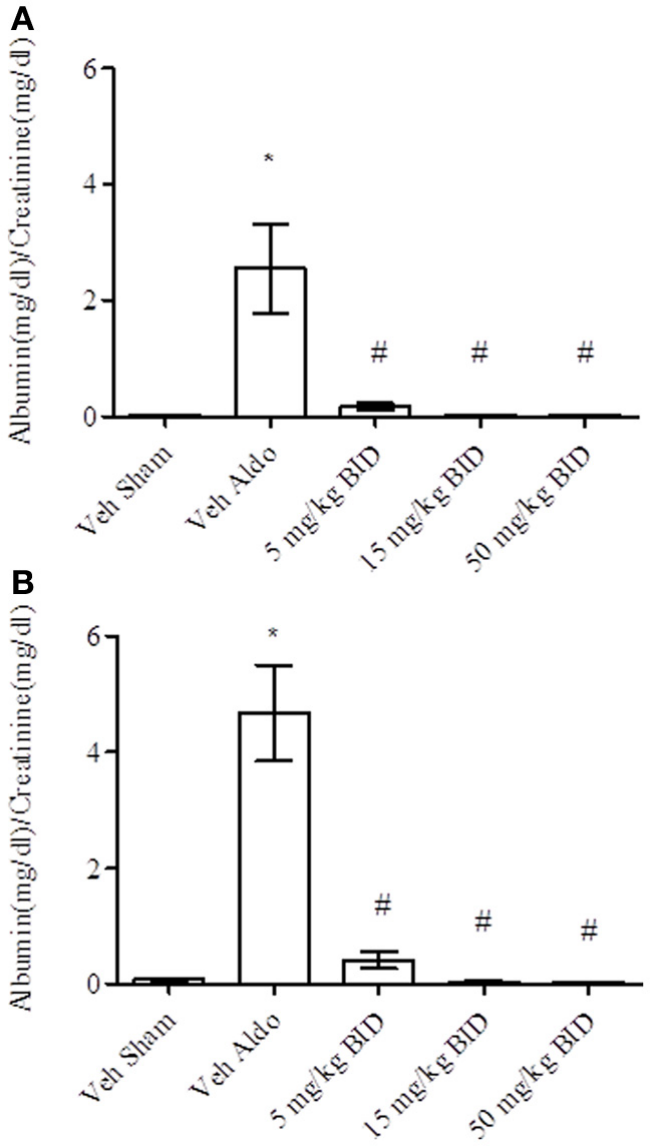
**Effect of PF-03882845 on UACR on day 14 (A) and day 26 (B).** Data are represented as mean ± standard error of the mean. See Materials and Methods for statistical analysis. ^*^Indicates a significant difference from the vehicle sham (Veh Sham) group; ^#^Indicates a significant difference from the vehicle + aldosterone (Veh Aldo) group.

**Figure 3:**
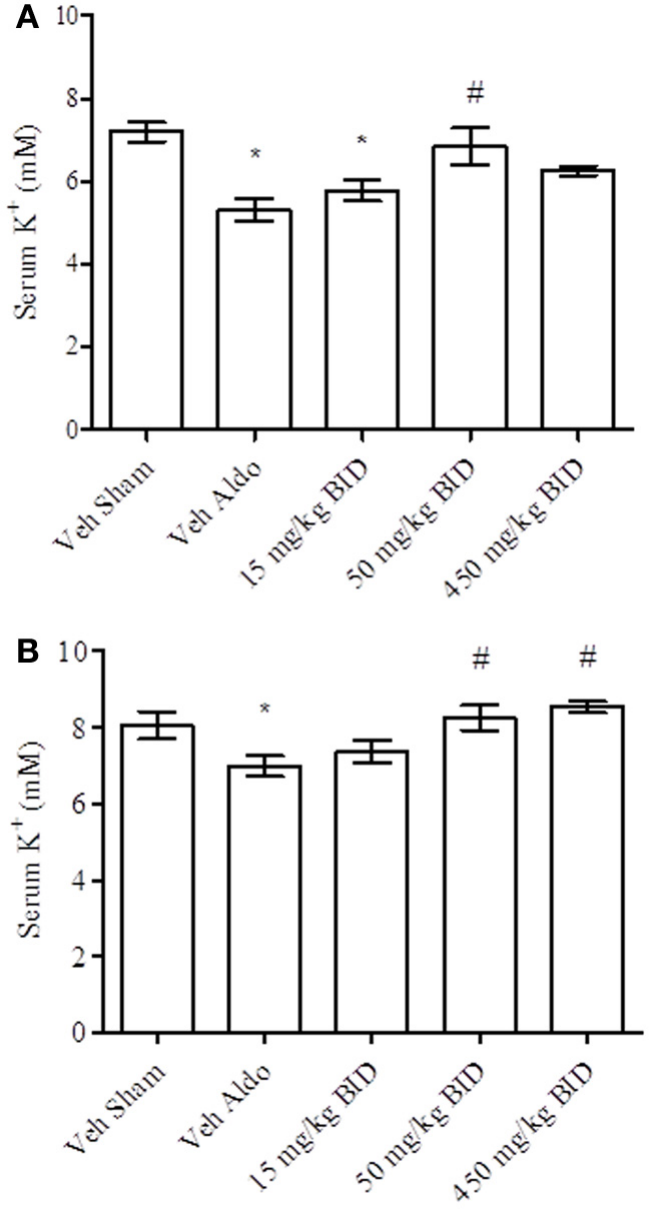
**Effect of eplerenone on serum K^+^ on day 14 (A) and day 26 (B).** Data are represented as mean ± standard error of the mean. See Materials and Methods for statistical analysis. ^*^Indicates a significant difference from the vehicle sham (Veh Sham) group; ^#^ Indicates a significant difference from the vehicle + aldosterone (Veh Aldo) group.

**Figure 4:**
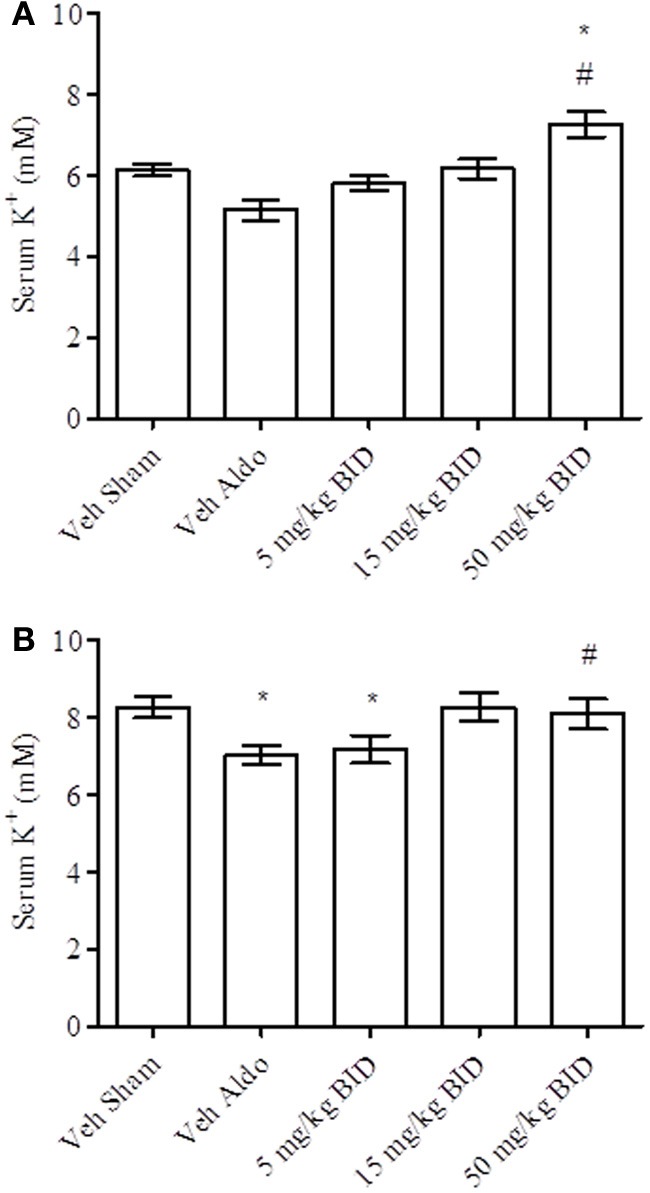
**Effect of PF-03882845 on serum K^+^ on day 14 (A) and day 26 (B).** Data are represented as mean ± standard error of the mean. See Materials and Methods for statistical analysis. ^*^Indicates a significant difference from the vehicle sham (Veh Sham) group; ^#^Indicates a significant difference from the vehicle + aldosterone (Veh Aldo) group.

Baseline UACR levels were not different amongst groups. Aldosterone infusion increased UACR levels relative to the vehicle sham group in both Study A and Study B by Day 14 (Figures 1A, 2A, respectively). This effect was more pronounced by Day 26 (Figures 1B, 2B, respectively). The highest dose of eplerenone (450 mg/kg BID) prevented the increase on Days 14 and 26 of treatment (Figures 1A,B) while all doses of PF-03882845 were efficacious in blunting UACR elevations (Figures 2A,B). Aldosterone decreased serum K^+^ on Days 14 and 26 in Study A (Figures 3A,B) but only on Day 26 in Study B (Figures 4A,B). By Day 26, eplerenone prevented the aldosterone-induced decrease in serum K^+^ at the doses of 50 mg/kg and 450 mg/kg BID (Figure 3B). A similar effect was observed with PF-03882845 at the highest dose tested (50 mg/kg BID; Figure 4).

### PK-PD modeling of UACR and serum K^+^

**Figure 5:**
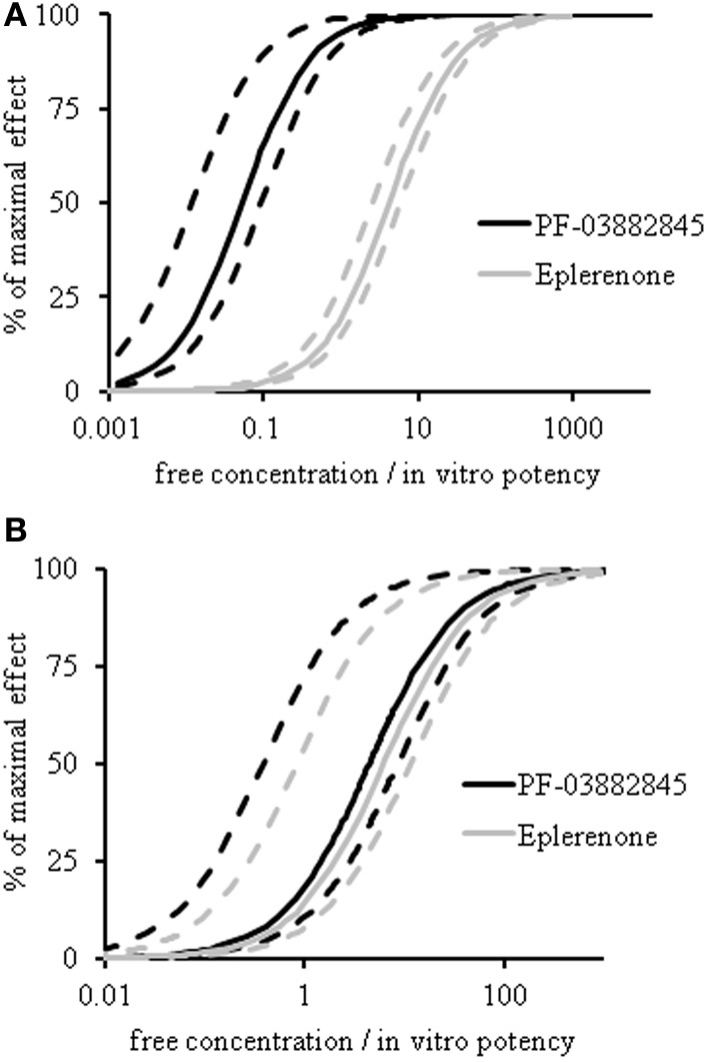
**PK-PD modeling of UACR and serum potassium.** Concentration-effect curves of eplerenone (gray lines) and PF-03882845 (black lines) for UACR **(A)** and serum K^+^ **(B)** following 27 days of treatment. Dashed lines indicate the 95% upper and lower confidence intervals. Data were modeled using an indirect response model. A double delta equation was used to express the pharmacodynamic response for serum K^+^.

Model estimated values of the EC_50_ for UACR and for serum K^+^ are depicted in Figure 5 and Table 1. Eplerenone and PF-03882845 resulted in overlapping concentration effect curves for serum K^+^ (Figure 5B) and the derived EC_50_ values were not significantly different (Table 1). By contrast, PF-03882845 was more potent than eplerenone in preventing the rise in UACR as observed in the modeled concentration-effect curves (Figure 5A). The resulting EC_50_ was 64-fold lower compared to eplerenone (Figure 5A and Table 1). The therapeutic index (TI), calculated as the ratio of EC_50_ for increasing serum K^+^ to the EC_50_ for suppressing UACR, was 1.47 for eplerenone and 84 for PF-03882845 (Table 1).

**Table 1:** **Chronic effect of PF-03882845 and eplerenone on UACR and serum K^+^**.

**Compound**	**Endpoint**	**EC_50_ nM (Total conc)**	**95% CI lower limit**	**95% CI upper limit**	**fEC_50_ nM (corrected for for protein binding)^a^**	**fEC_50_/*in vitro* potency^b^**	**TI (95% CI)^c^**
PF-03882845	Serum K^+^	874	75.5	1671	3.32	4.43	84.0
	UACR	10.4	2.36	18.5	0.0395	0.0527	(4.1–710)
Eplerenone	Serum K^+^	988	134	1843	672	6.16	1.47
	UACR	671	411	932	456	4.19	(0.14–4.5)

Data expressed as EC_50_ with 95% confidence interval (CI).

a fEC_50_: EC_50_ multiplied by fraction unbound: PF-03882845: 0.0038; Eplerenone: 0.68.

b In vitro potency: PF-03882845: 0.75 nM; Eplerenone: 109 nM.

c Therapeutic index (TI) calculated as the ratio of “EC_50_/in vitro potency” for serum K^+^ to “EC_50_/in vitro potency” for UACR.

### Effect of PF-03882845 and eplerenone on collagen IV staining in kidney

**Figure 6:**
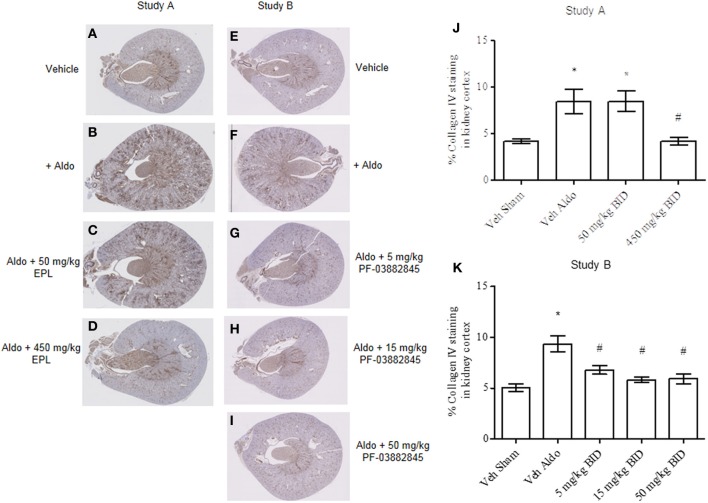
**Representative images of collagen IV immunohistochemical localization in kidney cortex.** Study A, vehicle sham **(A)**, vehicle + aldosterone **(B)**, 50 mg/kg BID eplerenone **(C)**, and 450 mg/kg BID eplerenone **(D)**, groups following 27 days of treatment; and Study B, kidney cortex from sham vehicle **(E)**, vehicle + aldosterone **(F)**, 5 mg/kg BID PF-03882845 **(G)**, 15 mg/kg BID PF-03882845 **(H)**, and 50 mg/kg BID PF-03882845 **(I)** following 27 days of treatment. Data expressed as % collagen IV staining in kidney cortex and represented as mean ± standard error of the mean: Study A **(J)** and Study B **(K)**. See Materials and Methods for statistical analysis. ^*^Indicates a significant difference from the vehicle sham (Veh Sham) group; ^#^Indicates a significant difference from the vehicle + aldosterone (Veh Aldo) group.

Continuous infusion with aldosterone significantly increased collagen IV staining in kidney cortex (Figures 6B,F) vs. the vehicle sham group (Figures 6A,E) in Study A and Study B, respectively. Eplerenone prevented the increase in collagen IV staining at the 450 mg/kg BID dose only (Figures 6C,D,J) while all three doses of PF-03882845 were efficacious (Figures 6G–I, 6K).

### Effect of PF-03882845 and eplerenone on gene expression in kidney

In studies A and B, aldosterone significantly increased mRNA expression of collagen 4a1, transforming growth factor β-1 (*Tgf-β 1*), interleukin-6 (*Il-6*), intercellular adhesion molecule-1 (*Icam-1*), monocyte chemoattractant protein-1 (*Mcp-1*), and osteopontin (*Spp1*) in the kidney on Day 27. This was prevented by PF-03882845 at all doses tested (Table 2), while eplerenone prevented the rise in these genes only at the highest dose tested (4.0 mg/kg BID) (Table 3).

**Table 2:** **Effect of PF-03882845 on relative gene expression in kidney on day 27**.

**PF-03882845**	**Veh sham**	**Veh aldo**	**PF-03882845 5 mg/kg BID**	**PF-03882845 15 mg/kg BID**	**PF-03882845 50 mg/kg BID**
*Tgf-1β*	0.95 ± 0.039	1.7 ± 0.14^*^	1.0 ± 0.088^ȧ^	0.93 ± 0.092^ȧ^	0.89 ± 0.071^ȧ^
*Col4a1*	0.92 ± 0.051	1.5 ± 0.11^*^	1.0 ± 0.099^ȧ^	1.1 ± 0.12^ȧ^	0.94 ± 0.059^ȧ^
*Il-6*	1.0 ± 0.14	6.6 ± 0.68^*^	1.6 ± 0.29^ȧ^	1.1 ± 0.18^ȧ^	1.2 ± 0.18^ȧ^
*Icam-1*	1.1 ± 0.050	1.8 ± 0.14^*^	1.1 ± 0.077^ȧ^	1.2 ± 0.12^ȧ^	1.2 ± 0.056^ȧ^
*Mcp-1*	0.96 ± 0.089	1.8 ± 0.26^*^	0.95 ± 0.082^ȧ^	0.64 ± 0.070^ȧ^	0.76 ± 0.090^ȧ^
*Spp1*	0.92 ± 0.11	11 ± 1.9^*^	1.3 ± 0.23^ȧ^	0.73 ± 0.081^ȧ^	0.60 ± 0.20^ȧ^

Values are mean relative quantitation ± standard error of the mean, n = 8–10/group. One-Way ANOVA with Tukey's post-hoc analysis was performed.

* Significantly different from vehicle sham (Veh sham);

∧ significantly different from vehicle + aldosterone (Veh aldo).

**Table 3:** **Effect of eplerenone on gene expression in kidney on day 27**.

**Eplerenone**	**Veh sham**	**Veh aldo**	**Eplerenone 15 mg/kg BID**	**Eplerenone 50 mg/kg BID**	**Eplerenone 4.0 mg/kg BID**
*Tgf-1β*	1.1 ± 0.071	1.9 ± 0.20^*^	1.7 ± 0.12	1.6 ± 0.20	1.2 ± 0.039^∧^
*Col4a1*	1.0 ± 0.050	1.6 ± 0.16^*^	1.4 ± 0.087^*^	1.2 ± 0.057	1.1 ± 0.050^∧^
*Il-6*	1.1 ± 0.25	6.3 ± 1.5^*^	5.0 ± 0.89^*^	5.5 ± 1.4	3.5 ± 1.5
*Icam-1*	1.2 ± 0.047	1.9 ± 0.18^*^	1.9 ± 0.20^*^	1.8 ± 0.25	1.2 ± 0.060^∧^
*Mcp-1*	1.1 ± 0.10	2.2 ± 0.25^*^	2.6 ± 0.56^*^	2.6 ± 0.54^*^	1.4 ± 0.26
*Spp1*	2.0 ± 0.33	46 ± 11^*^	25 ± 5.3^*^	30 ± 9.7^*^	3.2 ± 0.75^∧^

Values are mean realtive quantitation ± standard error of the mean, n = 10–11/group. One-way ANOVA with Tukey's post-hoc analysis was performed.

* Significantly different from vehicle sham (Veh sham);

∧ Significantly different from vehicle + aldosterone (Veh aldo).

### Effect of PF-03882845 and eplerenone on plasma osteopontin

Aldosterone increased plasma osteopontin levels in Study A (vehicle: 93.6 ± 16.4 ng/ml; vehicle + aldosterone: 345 ± 205 ng/ml (mean ± SEM)) and this effect was blunted by eplerenone at the 450 mg/kg BID dose (62.2 ± 7.98 ng/ml), while the doses of 15 or 50 mg/kg BID were not different from the vehicle + aldosterone group (eplerenone 15 mg/kg BID: 204 ± 35.1; eplerenone 50 mg/kg BID: 234 ± 85.6 ng/ml). In study B, aldosterone increased plasma osteopontin levels (vehicle: 87.3 ± 13.9 ng/ml; vehicle + aldosterone: 494 ± 90.1 ng/ml). This effect was suppressed at all doses of PF-03882845 (PF-03882845.5 mg/kg BID: 108 ± 13.5, 15 mg/kg BID: 77.1 ± 15.7 and 50 mg/kg BID: 82.4 ± 17.0 ng/ml, respectively).

### Acute effect of PF-03882845 and eplerenone on urinary Na^+^/K^+^ ratio

**Figure 7:**
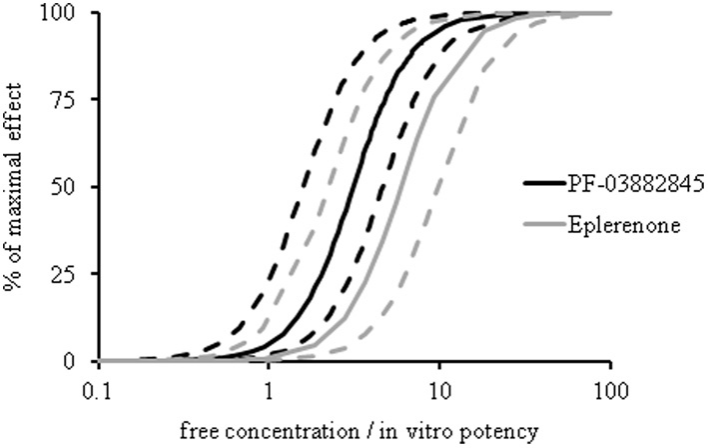
**Effect of eplerenone and PF-03882845 on urinary Na^+^/K^+^ ratio.** SD rats received single doses of 0, 5, 30, or 3.0 mg/kg eplerenone or 0, 3, 10, or 30 mg/kg PF-03882845 and urinary Na^+^/K^+^ was assessed at 0–2, 2–4, and 4–7 h post-dose. Both compounds yielded analogous concentration-effect curves for urinary Na^+^/K^+^. Data were expressed as free concentration normalized to *in vitro* IC50 (0.75 nM for PF-03882845 and 109 nM for eplerenone in a serum free assay). Data were modeled using an indirect response model. Dashed lines indicate the 95% lower and upper confidence boundary limits.

Urinary Na^+^/K^+^ ratio was assessed in SD rats following single doses of PF-03882845 or eplerenone. Concentration-effect curves were generated using an indirect response model. In agreement with the chronic effects of these compounds on serum K^+^ levels, the EC_50_ values for the acute effect on urinary Na^+^/K^+^ ratios were not different when corrected for the respective free fraction and *in vitro* potency (PF-03882845 (mean [95% confidence interval]: 3.10 [1.58–4.62]) and eplerenone (5.92 [2.22–9.63]) (Figure 7).

## Discussion

We investigated whether PF-03882845, a novel non-steroidal MR antagonist, differentiates from traditional steroidal MR antagonists, such as eplerenone. Our findings indicate that PF-03882845 exhibits a greater TI, calculated as the ratio of the EC_50_ for increasing serum K^+^ to the EC_50_ for UACR lowering, compared to eplerenone. Thus, our results support the development of PF-03882845 for the treatment of diabetic nephropathy, a condition in which the patient is particularly at risk for hyperkalemia.

Aldosterone binding to the MR mediates sodium and water retention in exchange for K^+^ secretion in the renal tubules. These effects appear to be downstream of the serum- and glucocorticoid-inducible kinase-1 (SGK1) (Lang et al., 2006; Terada et al., 2008). Indeed, aldosterone has been demonstrated to stimulate *Sgk1* mRNA expression (Naray-Fejes-Toth et al., 1999) and phosphorylation (Flores et al., 2005) in cortical collecting duct cells. Activated SGK1 inhibits turnover of the epithelial sodium channel (ENaC) thereby enhancing ENaC abundance at the plasma membrane (Butterworth et al., 2009) via phosphorylation and inhibition of the ubiquitinase Nedd4-2 which marks ENaC for proteasomal degradation (Flores et al., 2005). Sodium transport is also regulated by the With-No-K (lysine) kinase (WNK) family of kinases amongst which WNK4 has been demonstrated to phosphorylate and inhibit ENaC and the Na^+^ and Cl^−^ cotransporter (NCC) (Hoorn et al., 2009). In turn, WNK4 is phosphorylated and inhibited by SGK1 and by the “long form”-WNK1 (L-WNK1). Thus, aldosterone increases Na^+^ retention via SGK1 mediated phosphorylation/inhibition of WNK4, thereby relieving inhibition of ENaC and NCC (Hoorn et al., 2009; Vallon et al., 2009).

Aldosterone modulates K^+^ secretion via SGK1 upregulation of the renal outer medullary potassium (ROMK) channels in the thick ascending limb of Henle's loop, the distal tubule and in cortical collecting duct cells. L-WNK1 and WNK4 downregulate ROMK by stimulating clathrin-dependent endocytosis and this is abrogated by SGK1 mediated phosphorylation of Wnk4 (Welling and Ho, 2009). In addition to effects on ENaC and ROMK, aldosterone increases K^+^ secretion in exchange for sodium uptake via the basolateral Na^+^/K^+^ ATPase (Djelidi et al., 2001).

In the present study, aldosterone treatment of uninephrectomized rats depressed serum K^+^ levels as anticipated. In contrast, treatment with the MR antagonists blunted this effect, likely through preventing aldosterone-induction of ENaC, ROMK and the Na^+^/K^+^ ATPase. PK-PD modeling demonstrated that PF-03882845 and eplerenone were equipotent for increasing serum K^+^ levels relative to the vehicle + aldosterone group and for increasing the urinary Na^+^/K^+^ ratio following single doses in normal SD rats. Effects of MR antagonists on urinary Na^+^/K^+^ ratio have been well-characterized in rodents as well as humans (McInnes et al., 1982). Early on, Kagawa demonstrated blockade of mineralocorticoid activity in adrenalectomized rats by measuring urinary Na^+^/K^+^ ratio (Kagawa, 1960). This method was further simplified by Brandish et al. who revealed similar findings in rats with intact adrenal glands (Brandish et al., 2008). We have previously demonstrated translatability of this biomarker from rat to humans using preclinical and clinical data generated with eplerenone (Eudy et al., 2011). Our present findings extend these results by demonstrating similar concentration effect curves for urinary Na^+^/K^+^ ratio generated in normal SD rats following single doses of MR antagonists, and for serum K^+^ in an animal model of nephropathy following chronic exposure to the compounds. Thus, urinary Na^+^/K^+^ ratio in a healthy state appears to be a useful biomarker for assessing the risk of hyperkalemia in a renally compromised condition.

Interestingly, PK-PD modeling revealed that PF-03882845 was more potent than eplerenone in lowering UACR despite equipotent effects on serum potassium, thereby yielding a 57-fold greater therapeutic index against hyperkalemia compared to eplerenone. Albuminuria, a widely used surrogate biomarker of renal damage, has been previously demonstrated to be decreased by treatment with MR antagonists in patients with nephropathy (Sato et al., 2003; Epstein et al., 2006) and rodents with renal impairment (Blasi et al., 2003), supporting its modulation by aldosterone. Our results agree with these findings, and further highlight the potential of PF-03882845 in preventing/reducing renal damage, while posing less risk for hyperkalemia compared to a traditional MR antagonist. Concurrent with its effect on UACR, PF-03882845 was more potent than eplerenone in suppressing collagen IV gene and protein expression in kidney cortex. Collagen IV has been associated with glomerulosclerosis in experimental models (Floege et al., 1992; Bergijk et al., 1998; Lee et al., 1998) and humans (Kim et al., 1991; Tamsma et al., 1994; Esposito et al., 1996) with kidney disease. Renal biopsies from patients with diabetic nephropathy indicated a clear increase in α 2 type IV collagen gene and protein in glomeruli compared to controls (Adler et al., 2000). Importantly, aldosterone has been shown to stimulate collagen synthesis in rat renal fibroblasts (Nagai et al., 2005) and aldosterone infusion stimulated collagen III gene expression as assessed by *in situ* hybridization (Blasi et al., 2003). We expand on these findings by demonstrating increases of collagen IV gene and protein expression in kidney cortex with aldosterone infusion, and its blunting by the novel MR antagonist PF-03882845. Furthermore, our data indicate a greater potency of PF-03882845 compared to eplerenone for suppressing aldosterone-induced expression of proinflammatory and profibrotic genes in the kidney such as *Tgf-β*, *Il-6, Icam-1*, *Mcp-1 and Spp1*. ICAM-1 has been implicated in glomerular inflammation (Riser et al., 2001). Il-6 has been shown to upregulate *MCP-1* in human mesangial cells (Coletta et al., 2000). The latter was upregulated in urine of patients with diabetic nephropathy (DN) (Wada et al., 2000) and was decreased by spironolactone treatment (Takebayashi et al., 2006) suggesting regulation by the MR. TGF-β 1 has been demonstrated to reduce collagenase production thereby leading to accumulation of extracellular matrix components (Wolf, 2006). Importantly, Tgf-β 1 is increased in plasma of type 2 diabetics (Pfeiffer et al., 1996) and urine of type 2 diabetics with clinical diabetic nephropathy (Bertoluci et al., 2006) further supporting its role in the pathogenesis of this disease. In accordance with our findings, aldosterone infusion has been shown to increase TGF-β 1 protein expression in rat kidney (Juknevicius et al., 2004) and both TGF-β 1 and aldosterone independently stimulated connective tissue growth factor (CTGF), an inducer of renal fibroblast growth and synthesis of extracellular matrix proteins (Yokoi et al., 2001). In the present studies, aldosterone infusion also resulted in an increased intra-renal gene expression of osteopontin (*Spp1*), an extracellular matrix protein, chemokine and growth factor recently shown to be regulated by the MR in renal mesangial cells (Gauer et al., 2008). This effect was prevented more potently by PF-03882845 as compared to eplerenone. Importantly, plasma levels of osteopontin mirrored these findings. These results further support a greater potency of PF-03882845 vs. eplerenone in preventing renal damage as plasma levels of osteopontin have been shown to correlate with progression of diabetic nephropathy (Yamaguchi et al., 2004).

While our results clearly indicate a greater potency of PF-03882845 in reducing UACR compared to eplerenone, leading to an increased TI against hyperkalemia, mechanisms underlying these findings are yet to be determined. At least 3 hypotheses may explain the present data. First, PF-03882845 may differentially affect coactivator recruitment in different cell types leading to a greater repression of profibrotic pathways. Second, a greater uptake of PF-03882845 relative to eplerenone in cell types involved in filtration and fibrosis such as mesangial cells and/or podocytes compared to epithelial cells that regulate electrolyte balance could account for these findings. However, to-date, no evidence of active uptake of PF-03882845 into cells has been demonstrated. Third, the increased potency of PF-03882845 against fibrosis may be driven through off-target effects. Recently, aldosterone has been demonstrated to elicit effects on vascular smooth muscle cells at physiological concentrations through the GPR30 receptor (Gros et al., 2011). Further work is warranted to elucidate these findings.

In conclusion, PF-03882845, a novel non-steroidal MR antagonist, appears to present less of a risk for hyperkalemia while maintaining efficacy in preventing renal damage in a rodent model of nephropathy. Should these results translate into humans, PF-03882845 may present a safe and efficacious alternative for the treatment of chronic kidney disease.

### Conflict of interest statement

The authors are or were previously employed by Pfizer Inc, and possibly own stock options from Pfizer Inc. The authors and editor declare that while the author C. M. Boustany-Kari and the editor R. Fryer are currently employed by Boehringer-Ingelheim Pharmaceuticals, Inc., USA, there has been no conflict of interest during the review and handling of this manuscript.
